# Accuracy of ultrasound, magnetic resonance imaging and intraoperative frozen section in the diagnosis of ovarian tumours: data from a London tertiary centre

**DOI:** 10.1038/s44276-024-00068-4

**Published:** 2024-07-02

**Authors:** Sian Mitchell, Joseph Gleeson, Mansi Tiwari, Frances Bailey, Jonathan Gaughran, Gautam Mehra, Mustafa Zelal Muallem, Ahmad Sayasneh

**Affiliations:** 1 Guy’s and St Thomas’s NHS foundation trust, London, UK; 2 Department of Gynaecological Oncology, Guy’s and St Thomas’ NHS Foundation Trust, London, UK; 3 Centre for Oncological Surgery, Charité Medical University of Berlin, Berlin, Germany; 4 Faculty of Life Sciences & Medicine at Guy’s, The School of Life Course Sciences, King’s College London, London, UK

## Abstract

**Background:**

Ovarian cancer has the worst prognosis among all gynaecological cancers. The pre-operative and intraoperative diagnosis of ovarian tumours is imperative to ensure the right operation is performed and to improve patients’ outcomes.

**Methodology:**

A retrospective review of cases with a confirmed histological diagnosis of ovarian cases was undertaken from January 2017 to December 2021. Comparison was undertaken between this final diagnosis and the pre-operative ultrasound, MRI and frozen section (FS) to assess diagnostic accuracy of each. In the ultrasound cases, the level of the examiner was collected. Statistical analysis was performed using Stata MP v17.0 software (USA, 2023).

**Results:**

In total, 156 ovarian masses were examined by FS. In the histopathological examination, 123/156 of these tumours were epithelial tumours. Pre-operative US subjective impression was made in 63/156 cases and preoperative MRI subjective impression was made in 129/156 cases. For benign, borderline and malignant tumours, FS demonstrated a sensitivity of 90.8% (95%CI:81.9–96.2), 86.8% (95%CI:71.9–95.6) and 97.6% (95%CI:87.4–99.9) respectively. Ultrasound’s sensitivities were 95.2% (95%CI:76.2–99.9), 20% (95%:4.33–48.1), 57.1% (95%CI:28.9–82.3) and MRI’s sensitivities were 100% (95%CI:80.5–100), 31.5% (95%CI:19.5–45.6) and 63.2% (95%CI:46–78.2) respectively.

**Conclusions:**

FS remains an accurate tool for diagnosing ovarian malignancy. However, across both imaging modalities and FS, the diagnosis of borderline ovarian tumours remains challenging.

## Background

Ovarian cancer has the worst prognosis among all gynaecological cancers and is often diagnosed at advanced, less curable stages (stage 3 or 4). It carries significant morbidity and mortality with the 5-year survival for stage 4 disease being 13.4% [1]. The most common histological subtype is epithelial ovarian cancer and these account for approximately 80–85% of all ovarian cancers [2]. When the disease is diagnosed at stage 1, the 5-year survival rate is greater than 90%, however a standardized screening tool for ovarian cancer that significantly reduces death from the disease does not currently exist within the UK [1, 3]. It is therefore essential that there is accurate pre-operative characterisation of ovarian tumours to improve patient’s outcomes [4].

Accurate assessment of ovarian masses by ultrasound is of great importance. It is often the first line imaging modality to assess adnexal masses and subjective assessment by expert ultrasound examiners has demonstrated excellent results in distinguishing between benign and malignant masses [4–7]. The diagnostic accuracy of adnexal masses is operator dependent, however there are several prediction algorithms, with excellent results in practice to aid with triaging and diagnosis [8–11].

Although Magnetic Resonance Imaging (MRI) is a more expensive and not as readily available as ultrasound, it can add further information to those ovarian masses that are defined indeterminate by ultrasound [12, 13]. In previous studies, MRI has been shown to correctly identify malignant ovarian masses with an accuracy of 83–93% [14–16]. Different MRI sequences can add a different element of characterisation of ovarian masses, for example T2 weighted imaging has high soft tissue contrast resolution which can help in teratoma diagnosis. Scoring systems can further aid characterisation [17]. The ovarian adnexal reporting and data system (ORADS) MRI scoring system is frequently used as a malignancy risk stratification system with a sensitivity of 93.5% and specificity of 96.6% [18].

There are however continued challenges to the pre-operative diagnosis of ovarian masses and frozen sections can help to intraoperatively determine the malignant potential of an ovarian mass [19]. They can help determine and guide the operating surgeon on the extent of a surgical procedure including staging procedures [20]. This can avoid overtreatment in patients, which is of particular importance in cases of fertility preservation and can also avoid extensive surgical staging procedures which can carry a higher risk of visceral and vascular injury^13^.

Frozen section diagnosis is considered a reliable diagnostic method for assessing ovarian tumours with sensitivities reported between 90–96% [19, 21, 22].

The purpose of this retrospective study was to compare the diagnostic performance of ultrasound, MRI, and frozen section in the diagnosis of ovarian masses relative to the final histology of the masses.

## Methods

We conducted a retrospective observational study, between January 2018 to December 2021 and cases with known frozen section and final histology at Guy’s and St Thomas hospital, London were selected. All decisions for frozen section were made and discussed at our gynaeoncology multidisciplinary team (MDT) meeting. The decision for frozen section was made in cases where there was thought to be a possibility that there could be overtreatment due to unnecessary interventions or undertreatment that would require further treatment based on imaging alone.

The age and parity of all the patients were recorded. The carbohydrate antigen- 125 (ca-125) tumour marker data and the stage of the tumour was collected for the borderline and malignant cases.

All the specimens were processed and diagnosed by a consultant gynaecological pathologist; however, this was not the same pathologist for all cases. The results were reported back to the operating surgeon during the operation within 30 min of receiving the specimen.

All frozen section diagnosis were categorized as benign, borderline, and malignant, with the specific histology commented on if possible.

The MRI and US data that were performed at our tertiary gynaeoncology centre were used. Pre-operative MRI diagnosis was undertaken in 129 cases and ultrasound diagnosis in 63 cases.

The US, MRI and frozen section diagnosis was compared with the final paraffin histological section diagnosis in all cases.

For the US cases, the level of sonographer who performed the scan was recorded and grouped into level 1–3 examiners [23].

Approval of the study as a service improvement was granted by the audit department at Guys and St Thomas NHS trust. Formal ethical approval was not required.

### Statistics

Statistical analysis was performed using Stata MP v17.0 software (USA, 2023) [24]. To calculate the diagnostic performances of US, MRI, and frozen section in determining the benign, borderline and malignant status of an ovarian mass, performance measures including sensitivity, specificity, positive and negative likelihood rations were calculated according to the standard 2×2 method. Agreement was assessed using the Kappa co-efficient. The 95% confidence intervals were calculated for the sensitivities, specificities, and positive and negative predictive values.

## Results

From January 2017 – December 2021, a total of one hundred and fifty-six ovarian masses were examined by frozen section and by final histopathological examination. The median age of the patients in the study was 51, range 62 (80-18). The parity of the patients were varied with 56/156 nulliparous and 75/156 multiparous. Parity was not stated in 25/156.

In the final histopathological examination, 123/156 (78.8%) of tumours were epithelial tumours. Out of these cases, 55 were benign, 38 were borderline and 30 were malignant.

Sex cord stromal tumours accounted for 20/156 (12.8%) of tumours and 9 were benign and 11 were malignant. Benign germ cell tumours accounted for 10/156 (6.4%) of tumours. There were 3 tumours which were metastases (1 appendiceal, 2 colorectal). All the final histopathological examinations can be seen in Table 1.

**Table 1:** Distribution of all histological types according to final diagnosis.

Benign epithelial tumours	
Simple serous cyst	1
Serous cystadenoma	7
Serous cystadenofibroma	9
Serous fibroadenomas	2
Mucinous cystadenoma	21
Mucinous cystadenofibroma	1
Mucinous fibroadenoma	1
Mucinous cystadenoma and Brenner tumour	2
Seromucinous cystadenofibroma	2
Endometrioma	3
Endometriotic cystadenofibroma	1
Benign epithelial inclusion cyst	1
Benign corpus luteal cyst	2
Benign cyst, unclassified	1
Endometriosis with mucinous metaplasia	1
**Borderline epithelial tumours**	
Serous borderline tumour	17
Seromucinous borderline tumour	6
Mucinous borderline tumour	13
Borderline mucinous tumour with benign Brenner	2
**Malignant epithelial tumours**	
High grade serous carcinoma	7
Seromucinous carcinoma	1
Mucinous carcinoma	9
Mucinous carcinoma with serous mucinous features	1
Endometrioid ovarian ca	5
Clear cell carcinoma	6
Intra-epithelial carcinoma arising within borderline mucinous tumour	1
**Benign Sex cord stromal tumours**	
Fibroma	5
Fibrothecoma	3
Thecoma	1
**Malignant sex cord stromal tumours**	
Adult type granulosa cell tumour	9
Leydig cell tumour	1
Sertoli - leydig cell tumour	1
**Benign germ cell tumours**	
Mature cystic teratoma	4
Struma ovarii	5
Mature cystic teratoma with benign mucinous cystadenoma	1
**Mestatases**	
Appendiceal	1
Colorectal	2

For the 38 borderline cases, 14 of the cases did not have pre-operative ca-125 data. Of the remaining 24 cases, the median value for ca-125 was 15 u/ml and interquartile range was 38 (48-10). The Federation of Gynaecology and Obstetrics (FIGO) staging for the borderline tumours were as follows: stage 1 A were 26 cases, stage 1B were 2 cases, stage 1 C were 7 cases, stage 2 was 1 case, stage 3 were 2 cases and there were no cases for stage 4 disease.

For the 44 malignant cases, 11 of the cases did not have pre-operative ca-125 data. Of the remaining 33 cases, the median value for ca-125 was 44 u/ml and the interquartile range was 73 (85.5-12.5). The FIGO staging for the malignant cases were as follows: Stage 1 A were 21 cases, stage 1B was 1 case, stage 1 C were 8 cases, stage 2 were 3 cases, stage 3 were 7 cases and stage 4 was 1 case. 3 cases were metastases from other organs and therefore were not given FIGO staging.

Pre-operative ultrasound diagnosis was made in 63/156 patients (40.4%). Level 1 sonographers performed 41/63 (65.1%) of cases, Level 2 sonographers 8/63 (12.6%) cases and level 3 sonographers 14/63 (22.2%) cases. Ultrasound had an overall sensitivity and specificity of 95.2% (95% CI: 76.2–99.9) and 61.9% (95% CI: 45.6–76.4) for benign masses, 20% (95% CI: 4.33–48.1) and 79.2% (95% CI: 65–89.5) for borderline masses and 57.1% (95% CI: 28.9- 82.3) and 87.8% (95% CI: 75.2–95.4) for malignant masses (Table 2). Ultrasound showed poor agreement with histological cell type interpretation with a kappa coefficient of 0.29.

**Table 2:** Diagnostic performance of ultrasound, MRI and Frozen Section.

		Sensitivity (% (95% CI))	Specificity (% (95% CI))	Positive Predictive Value, PPV (95% CI)	Negative Predictive Value, NPV (95% CI)
Ultrasound diagnosis	Benign	95.2 (76.2–99.9)	61.9 (45.6–76.4)	55.6 (38.1–72.1)	96.3 (81–99.9)
	Borderline	20 (4.33–48.1)	79.2 (65–89.5)	23.1 (5.04–53.8)	76 (61.8–86.9)
	Malignant	57.1 (28.9–82.3)	87.8 (75.2–95.4)	57.1 (28.9–82.3)	87.8 (75.2–95.4)
MRI diagnosis	Benign	100 (80.5–100)	61.3 (51.5–70.4)	29.5 (18.5–42.6)	100 (94.7–100)
	Borderline	31.5 (19.5–45.6)	81.3 (70.7–89.4)	54.8 (36–72.7)	62.2 (51.9–71.8)
	Malignant	61.5 (44.6–76.6)	85.6 (76.6–92.1)	64.9 (47.5–79.8)	83.7 (74.5–90.6)
Frozen section diagnosis	Benign	90.8 (81.9–96.2)	93.8 (86–97.9)	93.2 (84.9–97.8)	91.5 (83.2–96.5)
	Borderline	86.8 (71.9–95.6)	97.5 (92.7–99.5)	91.7 (77.5–98.2)	95.8 (90.5–98.6)
	Malignant	97.6 (87.4–99.9)	95.6 (90.1–98.6)	89.1 (76.4–96.4)	99.1 (95–100)

The interpretation and the final histology of the 14 ovarian tumours that were classified by level 3 sonographers are shown in Table 3. There were 13/63 cases that were classified as uncertain on ultrasound by subjective impression. The histology for these can be seen in Table 4.

**Table 3:** This table demonstrates the subjective assessment of level 3 sonographers for the 14 ovarian tumours classified by them and the results of the final histology for the 14 ovarian tumours.

Ultrasound	Final histology		
	Benign	Borderline	Malignant
Benign	4	0	0
Borderline	2	1	2
Malignant	0	2	1
Uncertain	0	1	1

**Table 4:** Specific histology for ultrasound classified as uncertain by subjective impression.

Histology	Number (n)
Benign	
Mature cystic teratoma with benign mucinous cystadenoma	1
Mucinous cystadenoma and Brenner tumour	1
Benign corpus luteal cyst	1
Serous cystadenoma	1
Borderline	
Serous borderline tumour	3
Seromucinous borderline tumour	1
Mucinous borderline tumour	1
Malignant	
Endometrioid carcinoma of the ovary	1
Granulosa cell tumour	2
Seromucinous carcinoma	1

MRI was used to make a pre-operative impression in 129 patients. The sensitivity and specificity for benign tumours was 100% (95% CI: 80.5–100) and 61.3% (95% CI: 51.5–70.4) for benign tumours;31.5% (95% CI: 19.5–45.6) and 81.3% (95% CI: 70.7–89.4) for borderline tumours and for 61.5% (95% CI: 44.6–76.6) and 85.6% (95% CI: 76.6–92.1) for malignant tumours (Table 2). MRI showed agreement with histological cell type interpretation with a kappa coefficient of 0.34.

There were 18/129 cases that were classified as uncertain on MRI by subjective impression. The histology for these can be seen in Table 5.

**Table 5:** Specific histology for MRI classified as uncertain by subjective impression.

Histology	Number (n)
Benign	
Serous cystadenofibroma	1
Endometriotic cystadenofibroma	1
Serous cystadenoma	1
Serous fibroadenoma	1
Seromucinous cystadenofibroma	1
Serous cystadenofibroma	1
Mature cystic teratoma	1
Mucinous cystadenoma and Brenner tumour	1
Borderline	
Serous borderline tumour	1
Seromucinous borderline tumour	2
Mucinous borderline tumour	2
Malignant	
Serous carcinoma	2
Mucinous carcinoma	3

Frozen section was conducted on all 156 patients included in the study. Of these, there was discordance between the frozen section diagnosis and the final histopathological in 13/156 (8.3%) cases.

The sensitivity and specificity values were as follows: benign tumours:90.8% (95% CI: 81.9–96.2) and 93.8% (95% CI: 86–97.9); borderline tumours: 86.8% (95% CI: 71.9–95.6) and 97.5% (95% CI: 92.7–99.5) and malignant tumours: 97.6% (95% CI: 87.4–99.9) and 95.6% (95% CI: 90.1–98.6) (Table 2). Frozen section showed good agreement with histological cell type interpretation with a kappa coefficient of 0.88.

When borderline tumours were classified as malignant, ultrasound showed a sensitivity and specificity for malignant tumours of 58.6% (95%CI:38.9–76.5) and 70.6% (95%CI:52.5–84.9) respectively. The corresponding values for MRI were 62.4% (95% CI: 51.7–72.2) and 72.2 (95% CI: 54.8–85.8); and for frozen section were 93.8% (95% CI: 86–97.9) and 90.8% (95% CI: 81.9–96.2). All figures can be seen in Table 6.

**Table 6:** Diagnostic performance of each modality when borderline tumours are classed as malignant.

Malignant	Sensitivity (% (95% CI))	Specificity (% (95% CI))	Positive Predictive Value, PPV (95% CI)	Negative Predictive Value, NPV (95% CI)
Ultrasound	58.6 (38.9–76.5)	70.6 (52.5–84.9)	63 (42.4–80.6)	66.7 (49–81.4)
MRI	62.4 (51.7–72.2)	72.2 (54.8–85.8)	85.3 (74.6–92.7)	42.6 (30–55.9)
Frozen Section	93.8 (86–97.9)	90.8 (81.9–96.2)	91.5 (83.2–96.5)	93.2 (84.9–97.8)

## Discussion

### Summary of main results

Accurate characterization of ovarian masses, both pre-operatively with US and MRI and intraoperatively with frozen section is essential for precise management. Frozen section is vital for determining the malignant potential of an ovarian mass in a timely manner and is important for operative planning.

We performed diagnostic studies on the accuracy of ultrasound, MRI and frozen sections within our cohort of patients who had an intraoperative frozen section over a 5-year period.

Our results demonstrate that frozen section is an accurate method to appropriately characterise ovarian masses as benign, borderline and malignant intraoperatively with good diagnostic performance demonstrated throughout.

MRI and Ultrasound demonstrated good sensitivity for benign tumours, however ultrasound had poor sensitivity for malignant (57.1%) and borderline (20%) ovarian tumours.

#### Results in the context of published literature

A systematic review in 2011 demonstrated that if FS was benign or malignant, in 94% and 99% of the cases respectively, the histological diagnosis would remain the same, which is consistent with our study findings [25]. The accuracy of frozen sections for borderline tumours remains a major diagnostic challenge and studies have reported sensitivities as low as 0–50%. In all these studies, mucinous borderline tumours caused the greatest disagreement [26, 27].

It has been suggested that mucinous tumours are difficult to diagnose on frozen section as they are larger tumours with a mean tumour size 12 cm [28, 29]. In addition to this, they are more heterogenous masses with different histological subtypes and few areas of invasion in one mass when compared to their serous counterparts [30]. This would then require multiple samples to reach an accurate diagnosis. Misdiagnosis of borderline tumours is also related to <10% of borderline component and the experience of the pathologists [31].

In more recent studies, the rate of correlation between borderline tumours at frozen section and final histopathological diagnosis was 90.6% and this did not differ among different hospital settings or pathologists with different subspecialities [32].

This is similar to our dataset and could be due to the similarity in the distribution of serous and mucinous borderline tumours in both studies. Mucinous borderline tumours accounted for just under 50% of the borderline tumours analysed in this study.

In this study, 7 tumours were reported as benign at FS and later determined to be BOT or malignant. These were 3 borderline mucinous tumours and 4 malignant tumours (an appendiceal metastasis, ovarian intra-epithelial carcinoma, granulosa cell tumour and endometrioid carcinoma). The malignant cases were all stage 1 disease except for the metastases and 2 of these cases required further surgical treatment to stage the disease.

There was a false positive in one case. However, this patient was 70 years of age and had concurrent simple endometrial hyperplasia for which an MDT decision was made for a total abdominal hysterectomy, BSO and omental sampling. No other patients received extensive staging surgery unnecessarily.

MRI is a good preoperative tool for diagnosing ovarian masses and has particular use in determining sonographically indeterminate masses, however, its cost and lower availability prevents it’s use as the primary imaging modality for ovarian tumours [33, 34].

Our results for the sensitivity of MRI for diagnosis of benign tumours is consistent with the literature [35–37].

MRI has high contrast resolution and is helpful in detecting fat tissue and haemorrhagic areas as well as good sensitivity at detecting solid components and tumour septations [38]. This proves essential when assessing indeterminate tumours.

In the literature, borderline ovarian tumours are often misclassified on MRI and there is a range of sensitivities reported [39]. Bazot et al. reported the sensitivity and specificity of MRI for diagnosing borderline tumours as 45.5% and 96.1% respectively [37]. The sensitivity for serous vs mucinous BOT was 33.3% and 93.3% [37]. In this study a lower sensitivity was found for borderline and malignant ovarian masses, 31.5% and 63.2% respectively, in our dataset.

A systematic review has shown that MRI had a sensitivity of 92% and specificity of 85% for detecting borderline and invasive tumours [27]. For detecting malignancy alone, a sensitivity of 93% has been reported [14–16, 36, 40].

There have been several factors reported that can impact the diagnostic ability of an MRI. Vegetations and multilocularity were two factors could help determine serous from mucinous borderline tumours on MRI [41]. The size of the tumour is a significant factor in detection of tumours on MRI and it has been shown that there has been difficulty detecting lesions smaller than 2 cm [16].

It has further been reported that certain MRI techniques, such as dynamic and gadolinium contrast enhancement provides better resolution and characterization of these ovarian masses, improving sensitivity for detection [16, 42].

The heterogeneity between MRI imaging techniques, level of clinician reporting the scan, distribution of serous vs mucinous borderline tumours within the dataset and size of the tumours present could account for the variation in sensitivity, specificity and accuracy among different studies.

Ultrasound produces high resolution imaging of the ovaries and can distinguish simple ovarian cysts from complex ovarian masses [34]. Subjective assessment has an excellent sensitivity, as high as 96.7% for distinguishing between benign and malignant ovarian masses [7, 43–45]. Our study had good sensitivity for benign tumours but lower than expected sensitivity for malignant tumours.

Of the 63 cases that had a pre-operative US in our cohort, 14 of these had a malignant histopathological diagnosis. These included clear cell carcinoma, granulosa cell carcinoma and endometrioid carcinoma. In a large study describing the ultrasound features of clear cell carcinomas, it was reported that there was no specific ultrasound pattern for these tumours [46]. In other studies, granulosa cell tumours have been reported to have multiple sonographic appearances and endometrioid carcinoma are reported to be a rare diagnosis [47, 48]. Given the distribution of malignancies and taking into consideration that all pre-operative ultrasounds were not performed by only level 2 or 3 experts as in previous studies, this most likely accounts for the difference in sensitivities.

Specifically, when analysing the data for the level 3 examiners, the confirmed histological borderline and malignant tumours were only classified as borderline and malignant on US by the level 3 examiners. In clinical practice, all borderline and malignant ovarian tumours are triaged to a gynae-oncology cancer centre and so would receive the appropriate care. This is important to highlight as outcomes for ovarian cancer are better for patients when it is provided by trained gynaeoncologists in specialist centres [49]. Unfortunately, in this study it was difficult to directly compare the specific diagnostic performances at each level due to the small numbers.

Accurate preoperative ultrasound assessment of borderline tumours is imperative given the impact on patients’ fertility journeys; however, BOTs continue to pose a difficulty and the accuracy of ultrasound diagnosis is reported to be lower than for benign and malignant tumours [50].

Previous studies have reported a sensitivity of 44% and 69% for the subjective impression of BOTs on ultrasound diagnosis and a recent systematic review reports a sensitivity of 66% with a low false positive rate [50–52].

In this study, we reported a sensitivity of 20% for the ultrasound subjective assessment of ultrasound diagnosis. In addition to this, 5/13 unclassified tumours were BOTs. This is lower than reported in previous studies and this could be attributed to the expertise level of clinicians performing and interpreting the scans and the heterogeneity of the BOTs included in this study. This data does however support that sensitivity is lower than for benign or malignant tumours.

#### Strengths and weaknesses

To our knowledge, this is the first study that has compared the diagnostic performance of frozen section, MRI and US in the same cohort of patients, however it did have some limitations.

It was retrospective in nature, and this could be considered a weakness of the study. In addition to this, dissimilar from previous studies, the level of clinicians performing and interpreting the pre-operative imaging, in particular US, varied and was not solely level 3 examiners. This could have influenced the subjective impression reported; however results are generalizable to clinical practice where not all patients are scanned by level 3 clinicians.

We also acknowledge that there are a smaller number of preoperative ultrasound cases than there are MRI cases in this study. As a tertiary referral gynaeoncology centre, prior to referral to our unit, ultrasounds may be conducted at the patient’s base hospital. In our study, we only included ultrasounds which were performed within our trust. For future research and in comparisons of such data, an equal weighting of each imaging modality would be desirable.

We had chosen to report and include cases where the pre-operative imaging was unclassified. In practice, occasionally tumours may be subjectively described and given such diagnosis which had prompted further imaging and investigation.

#### Implications for practice and future research

To further improve early diagnosis of ovarian cancer on imaging modalities and improve outcomes for patients, further widespread training on ultrasound features of tumours for all grades of examiners is pivotal. Access to level 3 examiners in all hospital trust can be limited and upskilling all examiners is important.

From a research perspective, larger prospective studies including those comparing subjective impression of ultrasound of level 3 examiners to final histology are required to further build on this data.

In addition to this, further research is required to improve the diagnosis accuracy of pre-operative imaging modalities and in the era of increased research on computer assisted diagnosis in the diagnosis of ovarian tumours, this could reduce misdiagnosis and reduce intervention for many patients.

## Conclusion

To conclude, the results from frozen section and MRI remain good and there is still an important place for frozen section among pre-operative imaging. However, the diagnosis of BOTs remains challenging in all modalities.

## Data Availability

All data generated or analysed during this study are included in this published article.
